# Policies to Improve the Mental Health of People Influenced by COVID-19 in China: A Scoping Review

**DOI:** 10.3389/fpsyt.2020.588137

**Published:** 2020-12-11

**Authors:** Dan Qiu, Yilu Li, Ling Li, Jun He, Feiyun Ouyang, Shuiyuan Xiao

**Affiliations:** ^1^Department of Social Medicine and Health Management, Xiangya School of Public Health, Central South University, Changsha, China; ^2^Hunan Provincial Key Laboratory of Clinical Epidemiology, Changsha, China

**Keywords:** policy implementation, China, mental health policy, COVID-19, scoping review

## Abstract

**Background:** In response to the potentially concurrent mental health crisis due to the COVID-19 outbreak, there have been ongoing mental health policies put in place in China. This review aims to systematically synthesize the implemented national-level mental health policies released by the Chinese government during the COVID-19 outbreak, and summarize the implementation of those mental health policies.

**Methods:** Six databases and two websites were systematically searched, including published studies and gray literature published between December 1, 2019 and October 29, 2020.

**Results:** A total of 40 studies were included. Among them, 19 were national-level policies on mental health released by the Chinese government, and 21 studies reported data on the implementation of those mental health policies. Mental health policies were issued for COVID-19 patients, suspected cases, medical staff, the general population, patients with mental illness, and mental institutions. In the early stage of the COVID-19 epidemic, attention was paid to psychological crisis intervention. In the later stage of the epidemic, the government focused mainly on psychological rehabilitation. During the COVID-19 outbreak, more than 500 psychiatrists from all over China were sent to Wuhan, about 625 hotlines were notified in 31 provinces, several online psychological consultation platforms were established, social software such as TikTok, Weibo, and WeChat were used for psychological education, and many books on mental health were published. Responding quickly, maximizing the use of resources, and emphasizing the importance of policy evaluation and implementation quality were characteristics of the mental health policies developed during the COVID-19 outbreak. Challenges facing China include a low rate of mental health service utilization, a lack of evaluation data on policy effects, and no existing national-level emergency response system and designated workforce to provide psychological crisis interventions during a national emergency or disaster.

**Conclusions:** This review suggests that China has responded quickly and comprehensively to a possible mental health crisis during the COVID-19 outbreak, appropriate mental health policies were released for different members of the population. As the epidemic situation continues to change, the focus of mental health policies has been adjusted accordingly. However, we should note that there has been a lack of separate policies for specific mental health issues during the COVID-19 outbreak.

## Introduction

Over the past two decades, novel viruses have continued to emerge, the number of reported outbreaks of highly pathogenic or highly transmitted infectious diseases (such as SARS in 2003, H1N1 in 2009, MERS-CoV in 2012, and Ebola in 2014) has increased. At the end of 2019, a new type of infectious disease emerged, known as COVID-19 (1). As of November 10, 2020, over 49.7 million reported cases of COVID-19 and ~1.2 million deaths have been reported to the World Health Organization (WHO) (2). The outbreak of infectious diseases can spread rapidly, causing enormous losses to individual health, national economy, and social well-being (3).

The COVID-19 outbreak in China was shown to have a substantial negative impact on individuals' mental health, leading to clinical and sub-clinical disorders, such as acute stress disorder, depression, anxiety, post-traumatic stress disorder (PTSD), and other mental health symptoms (4–7). In response to the potentially concurrent mental health crisis, there have been ongoing mental health policies implemented in China, which has important ramifications for mental health systems and the patients they serve.

A mental health policy is a set of ideas or plans that are used as a basis for making decisions in mental health, it is a government statement that clarifies the values, principles, and goals of mental health (8). A mental health policy can be implemented at multiple levels, for example in the forms of mental health plans, programs, strategies, and legislation. If formulated and implemented properly, a mental health policy can become an important and powerful tool for countries to improve mental health and reduce the burden of mental disorders (8). Although some studies were conducted in the early stages of the COVID-19 epidemic, summarizing the mental health efforts of the Chinese government (9–11), many questions remain unanswered. This scoping review aims to present mental health policy developments during the COVID-19 outbreak in China and search for the answers to three questions that may be critical to policy making for other countries: (i) in different populations, is the focus of mental health policies different? (ii) what are the focuses of mental health policies at different stages of the epidemic? (iii) what is the implementation status of these mental health policies? To explore these questions, this review aims to systematically synthesize the national-level mental health policies released by the Chinese government during the COVID-19 outbreak through published research and gray literature, and summarize the implementation status of these mental health policies.

## Methods

A scoping review methodology (12, 13) was performed in this study according to the checklist of the PRISMA extension for scoping reviews (PRISMA-ScR) (14) (see Supplementary Table 1, Supplementary Material) and a methodology framework described by Arksey et al. (12) consisting of seven stages. The stages of the methodology framework undertaken were: (1) identification of research objectives; (2) reviewing data sources and search strategies; (3) study selection; (4) data extraction; (5) quality assessment for the included studies; (6) collating, summarizing, and analyzing outcome evidence; and (7) describing implications for further research.

### Research Objectives

- To identify the national-level mental health policies released by the Chinese government during the COVID-19 outbreak.
- To identify the focuses of mental health policies among different populations during the COVID-19 outbreak.
- To identify the focuses of mental health policies at different stages of the epidemic.
- To identify the implementation status of these mental health policies released by the Chinese government during the COVID-19 outbreak.

### Data Sources and Search Strategies

A systematic search was conducted in six databases and two websites.

Specifically, three databases including Web of Science, Pubmed, and CNKI were searched for published research. We set a restriction on the publication date, only studies published between December 1, 2019 and October 29, 2020 were searched for. See Additional File 1 for the details. The following search terms were used: “mental health policy” (including health policy, mental health policy, national plan, national program, etc.); “COVID-19” (including COVID-19, SARS-CoV-2, Coronavirus disease 2019, etc.); China (including China and Chinese). See Table 1 and the Supplementary Material for a full search strategy.

**Table 1:** Search strategies for the published studies.

**Search terms**	
Mental health policy	“national plan” (Title/Abstract) OR “national program” (Title/Abstract) OR “national strategy” (Title/Abstract) OR “legislation” (Title/Abstract) OR “law” (Title/Abstract) OR “national reform” (Title/Abstract) OR “health system” (Title/Abstract) OR “Health Policy” (Title/Abstract) OR “Mental health” (Title/Abstract) OR “Mental Health Policy” (Title/Abstract) OR “Mental Health Policies” (Title/Abstract) OR “effect of Health Policy” (Title/Abstract) OR “Policy Implementation” (Title/Abstract) OR “policy assessment” (Title/Abstract) OR “sychological assistant” (Title/Abstract)
COVID-19	“COVID-19” (Title/Abstract) OR “Coronavirus disease 2019” (Title/Abstract) OR “Covid 19” (Title/Abstract) OR “severe acute respiratory syndrome coronavirus 2” (Title/Abstract) OR “SARS-CoV-2” (Title/Abstract) OR “SARS-CoV” (Title/Abstract) OR “novel coronavirus” (Title/Abstract) OR “coronavirus” (Title/Abstract) OR “CoV-2” (Title/Abstract) OR “2019-nCoV” (Title/Abstract) OR “SARS COV2” (Title/Abstract)
China	“China” (Title/Abstract) OR “Chinese” (Title/Abstract)

Three databases including the National Science and Technology Library of China, the State Council Policy Document Database of China (http://www.gov.cn/index.htm), and the National Library of China and two websites including the Chinese Association for Mental Health (http://www.camh.org.cn/) and the Chinese Psychological Society (https://www.cpsbeijing.org/) were searched for gray literature. Keywords such as “policy,” “national plan,” “national program,” “COVID-19,” and “SARS-CoV-2” were combined with “mental health” in our search.

### Selection Criteria

A national-level mental health policy is defined in this review as a mental health policy released by the State Council of China and its direct departments, aimed at improving the mental health of people who were influenced by COVID-19.

Inclusion criteria
We included studies that fulfilled all the following criteria:

a) For mental health policy documents issued by the government

- Participants: people who were influenced by COVID-19 and lived in China.
- Intervention: any national-level policy interventions delivered to people influenced by COVID-19 in China, aimed at improving their mental health.

b) For studies reporting data on the implementation of mental health policies

- Participants: people who were influenced by COVID-19 and lived in China.
- Intervention: any national-level policy interventions delivered to people influenced by COVID-19 in China, aimed at improving their mental health.
- Outcome: implementation status of mental policy interventions delivered to people influenced by COVID-19 in China, the quantity of mental health services provided in response to these mental health policies (such as how many psychiatrists were sent to provide psychological crisis intervention, how many hotlines were conducted, etc.).
- Study design: no restriction
  Exclusion criteria
  We excluded studies if:
- The study was not in English or Chinese.
- The study was a theoretical study, describing standards that mental health policies are expected to meet.
- The study was discussing the process of policy formulation.
- The report was a protocol.

### Data Extraction

Identified records were first screened based on their titles and/or abstracts, and if they met the selection criteria, the full texts were obtained for further screening. The screening process was carried out independently by two authors (DQ and YLL). Data were extracted on date of publication, organizations, title of policy, and the main content of the policy independently by two reviewers (DQ and YLL).

### Data Synthesis

The qualitative data of the included studies were combined in a synthesis (15, 16). The results were grouped, where possible, by two analytical themes: (i) policies to improve the mental health of people during the COVID-19 outbreak and (ii) the implementation of mental health policies developed during the COVID-19 outbreak. Consensus was reached on discrepancies in data extraction through discussion.

### Quality Assessment

The methodological quality of the included studies was independently assessed by two reviewers (DQ and LL). Due to the varying study designs of included articles, checklists from The Joanna Briggs Institute (JBI) were utilized to assess content validity (17). See Supplementary Table 2 for details on the quality assessment.

## Results

**Figure 1:**
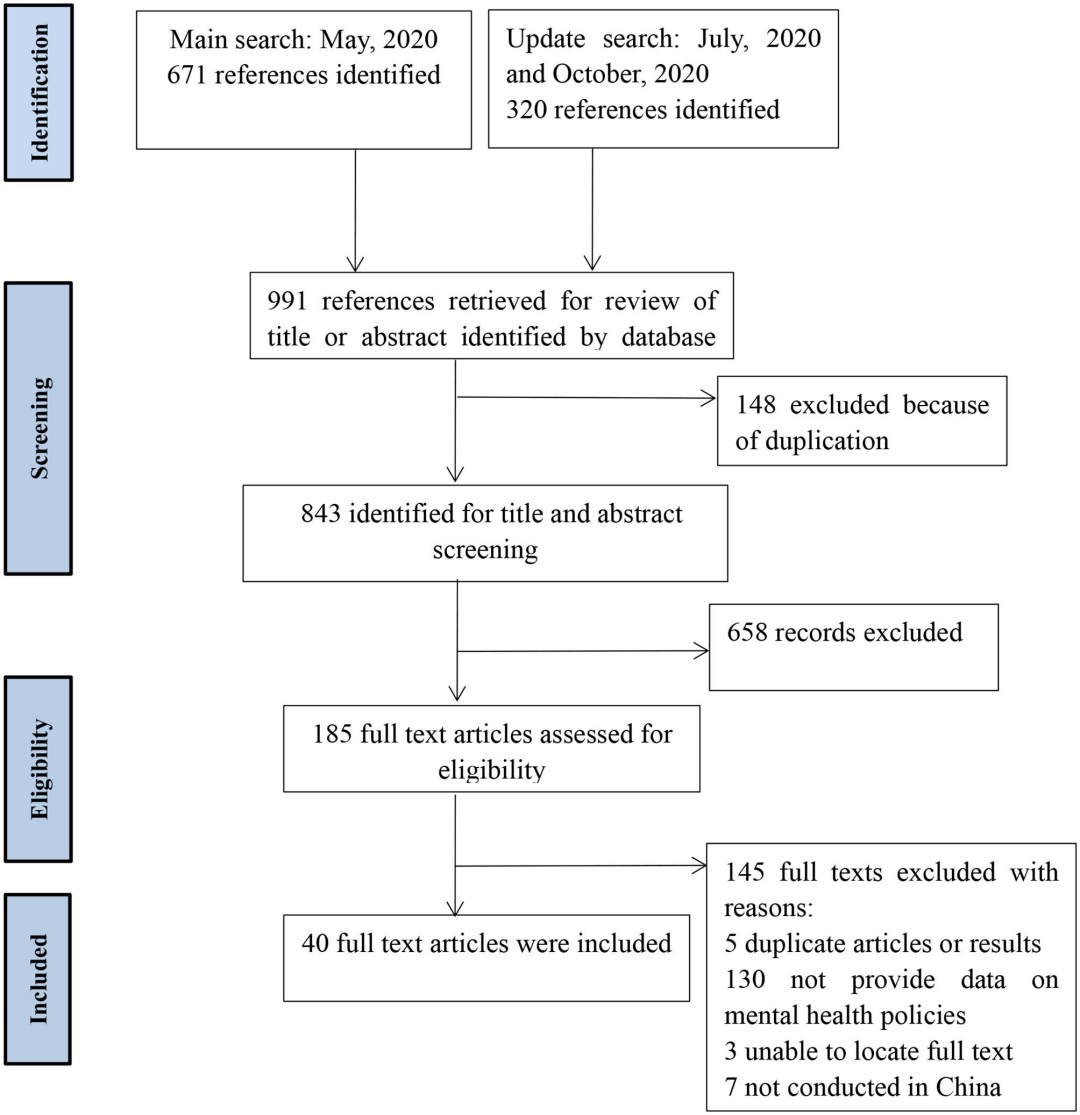
Flow of studies through review (57).

From the initially identified 991 records, 843 records were screened after duplicates were excluded. A total of 658 references were excluded based on the title or abstract, leaving 185 studies for further scrutiny. After screening the full texts, 145 studies were excluded. The 145 studies were excluded due to the following reasons: no data on mental health policies (*n* = 130); duplicate publications (*n* = 5); no full-text (*n* = 3); and not conducted in China (*n* = 7). Finally, 40 studies were included for analysis. See Figure 1 for the details.

### Characteristics of the Included Studies

From January to July 2020, the Chinese government has issued a total of 19 national-level policies related to mental health (18–36). Among them, there were eight policies related to patients with COVID-19 or suspected cases (18, 25–30, 36), seven were related to medical staff (18, 21, 24–27, 29), five were related to the general population [18–20, 29, 32[, two were related to teachers and students (19, 35), one was related to patients with severe mental disorders (22), two were related to mental health institutions (23, 31) and one was related to inward personnel (33). Besides, 18 studies (9–11, 37–54) reported data on the implementation of mental health policies developed during the COVID-19 outbreak. Of the 21 studies, 17 were of sound methodological quality, while the remaining articles were reported to have indeterminate quality, see Supplementary Table 3 for the details.

### Policies to Improve the Mental Health of People During the COVID-19 Outbreak

For patients with COVID-19 or suspected cases, the first guideline document was released on January 26, 2020. In this document (18), the National Health Commission of China suggested that all provinces in China invite psychologists and psychiatrists to organize psychological assistance teams and conduct psychological crisis interventions for patients in need. On March 5, three guideline documents for patients with COVID-19 or suspected cases were released by the Chinese government (25–27). Different psychological assistance programs were proposed for patients with different conditions. New psychological counseling work proposals were released on March 18 and April 7. Besides patients, these proposals paid more attention to the mental health of their family, friends, and colleagues (29, 30). Also, measures were taken to promote internet and psychosocial services, helping patients return to normal life. On March 4, the first version of the rehabilitation treatment proposal for discharged patients was released (28). All provinces in China were required to provide psychological rehabilitation intervention to discharged patients in need. The second version of the rehabilitation treatment proposal for COVID-19 patients was released on May 13, a more detailed psychological intervention plan was proposed for different types of mental health problems (36). Additionally, it was recommended that local governments organize social workers to conduct health education, eliminate community discrimination, and reduce patients' stigma.

For medical staff, the Chinese government proposed six related policies from January 26 to March 18. In January, the point of the policy was to provide emergency psychological crisis intervention for medical staff in need (18). In February, the list of online psychological assistance institutions for medical staff was released. The main target was to improve the protection measures of medical staff, improve their working environment, provide psychological assistance, and relieve their anxiety and stress (21, 24). Providing psychological education and skills training, organizing psychological support and emotional counseling services, and improving the self-help skills of medical staff were the main targets in March (25–27).

For the general population, the Chinese government released a total of six related policies from January 26 to April 14. Between January and March, the main targets of those policies were to provide internet/telephone-based emergency psychological crisis intervention or face-to-face psychological assistance for those people in need (18–20, 29). In April, the main targets changed to guiding people in medium-risk and high-risk areas to adapt to a closed life (32). For teachers and students, the Chinese government proposed a total of two related policies from January 28 to May 8. On January 28, the Ministry of Education required psychiatrists and psychologists in the education system to open psychological support hotlines and online counseling services, and provide online psychological crisis intervention services for teachers and students (19). In May, primary schools, secondary schools, and kindergartens in most areas reopened, the main targets changed to carrying out psychological education, psychological consultation, and creating psychological help hotlines for teachers and students (35). As the overseas epidemic become more serious, the Chinese government proposed measures against inward personnel in April, trying to provide psychological counseling and psychological intervention for inward personnel in need (33).

In order to prevent the spread of COVID-19 in mental health institutions and patients with severe mental disorders, the Chinese government proposed a total of three policies (22, 23, 31). Mental health institutions were suggested to strengthen hospital infection management, reserve protective equipment (such as masks) and disinfection materials, monitor the health status of staff and patients, and set up quarantine wards, etc. In addition, the proposal for “Building a National Psychosocial Service System-Key Tasks in 2020” was released (34). The government established specific evaluation criteria, added Wuhan as a new experimental city, and tried to comprehensively improve the mental health service level in Wuhan in a year. See Table 2 for the details.

**Table 2:** Summary of policies to improve the mental health of people during the COVID-19 outbreak.

**Date**	**Organizations**	**Policies/guidelines**	**Target population/institution**	**Main content on mental health**
January 26, 2020 (18)	National Health Commission of China Bureau of Disease Prevention and Control	“Principles of the Emergency Psychological Crisis Interventions for the New Coronavirus Pneumonia” was released	Patients with COVID-19; suspected cases; medical staff; relatives/friends of patients; the general population	All provinces in China were required to invite psychologists and psychiatrists to organize psychological assistance teams, and conduct psychological crisis interventions.
January 28, 2020 (19)	Ministry of Education	“Principles of Opening Psychological Support Hotlines and Network Counseling Services for the New Coronavirus Pneumonia in the Education System” was released	Teachers and students; the general population	Psychiatrists and psychologists in the education system were required to open psychological support hotlines and online counseling services, and provide online psychological crisis intervention services.
February 2, 2020 (20)	National Health Commission of China Bureau of Disease Prevention and Control	“Notice on Establishing Psychological Assistance Hotlines for the COVID-19 Outbreak” was released	The general public	All provinces in China were required to establish new hotlines, provide psychological support, psychological counseling, crisis intervention, and other services for different groups of people.
February 15, 2020 (21)	The State Council of China	“Measures to Improve the Working Conditions of First-Line Medical Staff and to Care for Their Physical and Mental Health” was released	Medical staff	All provinces in China were required to provide medical staff with online psychological support.
February 18, 2020 (22)	National Health Commission of China	“Notice on Strengthening the Treatment and Management of Patients with Severe Mental Disorders During the New Coronary Pneumonia Epidemic” was released	Patients with severe mental disorders	All provinces in China were required to strengthen the management and treatment of patients with severe mental disorders in hospitals and homes, preventing nosocomial infections in mental hospitals, and reducing the risk of accidents among patients.
February 25, 2020 (23)	National Health Commission of China	“Technical Proposal for the Prevention of New Coronary Pneumonia Outbreaks in Mental Health Institutions” was released	Mental health institutions	Mental health institutions in China were required to develop an emergency work plan, strengthen hospital infection management, reserve protective equipment (such as masks) and disinfection materials, monitor the health status of staff and patients, and set up quarantine wards, etc.
February 27, 2020 (24)	National Health Commission of China	“Guidelines for Psychological Assistance Hotline During the Prevention and Control of New Coronavirus Pneumonia” was released	Hotline staff	All provinces in China were required to conduct personnel training and provide hotline services in accordance with the guidelines.
March 4, 2020 (28)	National Health Commission of China	“Rehabilitation Proposal for Discharged Patients with New Coronary Pneumonia (draft)” was released	Discharged patients with COVID-19	All provinces in China were required to provide psychological rehabilitation intervention to discharged patients in need.
March 5, 2020 (25)	National Health Commission of China; Ministry of Civil Affairs	“Proposal for Psychological Assistance and Social Work Services in Cabin Hospitals” was released	Patients with COVID-19; medical staff	The psychological assistance team were required to provide psychological assistance in the cabin hospital according to this proposal.
March 5, 2020 (26)	National Health Commission of China; Ministry of Civil Affairs	“Proposal for Psychological Assistance and Social Work Services in Designate Hospitals” was released	Patients with COVID-19; medical staff	The psychological assistance team were required to provide psychological assistance in the designated hospital according to this proposal.
March 5, 2020 (27)	National Health Commission of China; Ministry of Civil Affairs	“Proposal for Psychological Assistance and Social Work Services for People in Quarantine” was released	Patients with COVID-19; medical staff	The psychological assistance team were required to provide psychological assistance for people in quarantine according to this proposal.
March 18, 2020 (29)	National Health Commission of China;	“Psychological Counseling Work Proposal for the New Coronavirus Pneumonia” was released	Patients with COVID-19; Suspected cases; medical staff / other related staff; relatives/friends of patients; the general population	All provinces in China were required to provide psychological counseling, psychological intervention, and other psychological services for key groups such as patients and their families, family members of deceased persons, and front-line workers, etc.
April 7, 2020 (30)	National Health Commission of China;	“Psychological Counseling and Social Work Service Proposal for Patients with New Coronary Pneumonia, Their Families, and People Were Quarantined” was released	Patients with COVID-19 and their families; people who were quarantined	All provinces in China were required to follow this proposal, conduct health education, promote internet and psychosocial services, help them return to normal life and work, and create a social environment of mutual care.
April 8, 2020 (31)	National Health Commission of China	“Technical Proposal for the Prevention of New Coronary Pneumonia Outbreaks in Key Places and Key Institutions” was released	Various public places; mental health institutions	Relevant departments were required to reserve protective equipment (such as masks) and disinfection materials, monitor the health status of staff, set up quarantine wards, and organize knowledge training on prevention of new coronary pneumonia, etc.
April 14, 2020 (32)	Ministry of Civil Affairs National Health Commission of China	“Proposal for Precise Community Prevention Service for the New Coronavirus Pneumonia” was released	The general population	All provinces in China were required to set up a community working group, establish a psychological assistance mechanism for community residents, and help them adapt to the closed management of the community.
April 21, 2020 (33)	National Health Commission of China Ministry of Civil Affairs Ministry of transport	“Psychological Counseling and Social Work Service Proposal for Inward Personnel” was released	Inward personnel	Conduct health education, promote internet and face-to-face psychosocial services, and provide psychological counseling and psychological intervention for people in need.
April 27, 2020 (34)	Ministry of Civil Affairs National Health Commission of China Ministry of Education	“Proposal for Building a National Psychosocial Service System-Key Tasks in 2020” was released	Wuhan city	Wuhan was added as a new experimental city, specific evaluation criteria was established, which tried to comprehensively improve the mental health service level in Wuhan in 2020.
May 8, 2020 (35)	Ministry of Education; National Health Commission of China	“Technical Guidelines for Prevention of New Coronavirus Pneumonia in Primary, Secondary Schools and Child Care Institutions” was released	Teachers; adolescents; children	Relevant schools were required to conduct mental health knowledge training, conduct psychological counseling, and announce a psychological hotline.
May 13, 2020 (36)	Ministry of Education; National Health Commission of China; National Healthcare Security Administration; National Administration of Chinese Medicine	“Rehabilitation Treatment Proposal for COVID-19 Patients with Major Dysfunction” was released	Discharged patients with COVID-19;	Relevant hospitals were required to conduct rehabilitation interventions for patients with emotional problems, cognitive changes, behavioral disorders, or physiological reactions due to psychological problems; communities were required to strengthen community publicity and reduce social discrimination.

### The Implementation of Mental Health Policies Developed During the COVID-19 Outbreak

In response to the first guideline document published on January 26, the Chinese Psychological Society and other psychological institutions organized the first round of personnel training on January 28 and released serval guidelines for hotline services between January 31 to February 6 (9, 20, 38, 50, 53). On February 7, the National Health Commission of China published a psychological adjustment guideline for the general public (49, 52). Between January and March, several books associated with COVID-19 and mental health were published (10, 40, 43). Such as “Guidelines for Public Psychological Self-Help of 2019-nCoV Pneumonia” and the “Handbook of Psychological Intervention for Government Departments, Enterprises, and Organizations.” Also, several platforms for psychological support were developed, those platforms developed online psychological self-help intervention systems, including interventions such as online cognitive behavioral therapy (41, 46, 47). Additionally, the Chinese Psychological Society published five lists of recommended hotline organizations, conducted three national surveys to investigate the mental health status of the general population, and provided references for the government to adjust its mental health policy in time (51).

At the end of February, more than 500 psychiatrists from all over China had been sent to Wuhan by several provinces to provide on-site psychological assistance for front-line medical staff and critically ill patients with COVID-19 (42, 45, 54). As of March 27, a total of 625 hotlines were notified in 31 provinces across China, with over 200,000 calls answered (39). A series of online mental health education programs on social media platforms, such as WeChat, Weibo, and Douyin (TikTok), have been developed and widely used for patients, medical staff, and the general public throughout all provinces in China (11, 43, 47). Besides, from January to June, the Chinese Psychological Society and other psychological institutions provided face-to-face psychological crisis intervention services for patients and medical staff in several hospitals in Wuhan according to the related policies (43, 44). Furthermore, some mental health institutions proposed a series of effective ward management strategies based on related policies and presented practical and resource-efficient models of psychological intervention for inpatients (48). See Table 3 for the details.

**Table 3:** Implementation of mental health policies developed during COVID-19 outbreak.

**Date**	**Organization**	**Implementation of policy**
January 28, 2020 (10)	Chinese Psychological Society Chinese Association of Mental Health Chinese Society of Psychiatry	Conducted first round of training for supervisors
January 30, 2020 (37)	Institute of Psychology, Chinese Academy of Sciences Chinese Psychological Society Ali Pay	National Crisis Intervention Platform for New Coronavirus Pneumonia was developed
January 31, 2020 (38)	Chinese Psychological Society	Published handbook for hotline organizations and volunteers “Guidelines on Providing Hotline Psychological Support for Disease Outbreak (draft)”
February 2, 2020 (19)	National Health Commission of China	Published guidelines for hotline organization, published list of organizations for psychological counseling
February 3, 2020 (10)	Chinese Psychological Society	Updated guidelines for hotline organization
February 6, 2020 (50, 53)	Chinese Psychological Society	Published ethics guidelines
February 7, 2020 (49, 52)	National Health Commission of China Bureau of Disease Prevention and Control	Published guidelines for the general public “Psychological Adjustment Guidelines for Coping with the New Coronavirus Pneumonia” was published
February 12, 2020 (10)	Chinese Psychological Society Chinese Association of Mental Health Chinese Society of Psychiatry	“Guidelines for Online Psychological Assistance Services during the Prevention and Control of Novel Coronavirus Pneumonia” was published
February 9, 2020 (10)	Chinese Society of Psychiatry Central Radio and television Station	Video of “Online Lessons of Psychological Intervention during the New Coronavirus Pneumonia Outbreak” was published
February 12, 2020 (10)	Peking University Sixth Hospital Beijing Anding Hospital Mental Health Center of Hubei province	Conducted second round of training for supervisors
February 12, 2020 (46, 47)	Wuhan University People's Hospital Peking University Sixth Hospital The First Affiliated Hospital of China Medical University Southern Hospital Beijing Anding Hospital, etc.	Platform for psychological support was developed. Online psychological self-help intervention systems, including online cognitive behavioral therapy were developed
February 2020 (9, 44, 45, 54)	National Health Commission of China/Several provinces in China	More than 500 psychiatrists from all over China were sent to Wuhan to provide on-site psychological assistance for front-line medical staff and critically ill patients;
February 24–June 4, 2020 (50)	Chinese Psychological society	Published five lists of recommended hotline organizations
March 27, 2020 (39)	32 provinces in China	A total of 625 hotlines were announced in 31 provinces in China, with over 200,000 calls answered
January–March 2020 (52)	Chinese Psychological society	The mental health status of the general population was investigated on January 31, February 6, and March 10. Data were provided for the adjustment of a national psychological assistance plan
January–March 2020 (38, 40, 43)	Chinese Psychological Society Chinese Association for Mental Health Chinese Society of Psychiatry, etc.	Several books associated with COVID-19 and mental health were published. Such as “Guidelines for Public Psychological Self-Help and Counseling of 2019-nCoV Pneumonia,” “Handbook of Psychological Intervention for Government Department”
May 2020 (47, 48)	Mental health institutions	Practical and resource-efficient models of psychological intervention were presented to inpatients
January 29–June 17, 2020 (43, 44)	Chinese Psychological society	From January to June, psychological crisis intervention services were provided for patients in several hospitals in Wuhan
January–March 2020 (48)	Mental health institutions	Some mental health institutions proposed a series of effective ward management strategies based on related policy
January–July 2020 (34, 44, 48, 52)	Government, universities, mental health institutions, social organization, Chinese Psychological society, etc.	Online mental health education with communication programs, such as WeChat, Weibo, and Douyin (TikTok), were widely used for medical staff and the general public throughout all provinces in China

## Discussion

### Key Findings

Our review suggests that China has responded quickly and comprehensively to the possible mental health crisis among different populations during the COVID-19 outbreak. In response to the COVID-19 outbreak, there have been ongoing measures and concerted efforts in China, resources were used as much as possible to implement these mental health policies. In the early stages of the epidemic, the focus of these mental health policies was to mobilize various resources and provide psychological crisis intervention for people in need. In the later stage, the focus of these mental health policies was on psychological rehabilitation intervention. Besides, the importance of providing online psychological counseling and improving people's self-help ability has been emphasized throughout the epidemic period. Psychiatrists from all over China were sent to Wuhan by several provinces to provide on-site psychological assistance. Mental health hotlines were quickly established across China and provided patients, medical staff, and the general public with counseling and psychological services. The telephone-based and internet-based programs were widely used to deliver mental health care services, and social media platforms (e.g., TikTok, WeChat, and Weibo) were widely utilized to share strategies, guidelines, and education programs for managing potential mental health problems. In addition, governments, academic groups, and medical institutions also published many self-help handbooks on psychological interventions related to COVID-19.

### Characteristics of Mental Health Policies During the COVID-19 Outbreak

Firstly, psychosocial interventions were organized very quickly to deal with the COVID-19 outbreak. On January 26, just 3 days after the lockdown implementation of Wuhan, the Chinese government published a guideline document for emergency psychological crisis interventions for the public (18). After this document was released, psychiatrists from all over China were immediately sent to Wuhan to provide psychological assistance, and several hotlines and internet-based platforms were developed for psychological counseling (10). However, some drawbacks of these policies should be noted. For example, who should deliver which type of intervention, for which group in need, and by which delivery mode were not specified in most policies (55). Also, we think there was a lack of separate policies for specific mental health issues during the COVID-19 outbreak. Such as PTSD, which is one of the most common mental health problems in a national emergency or disaster (56).

Another feature was the extensive use of mental health resources. Under the guidance of these mental health policies, cooperation between different institutions and organizations maximized the use of resources. Since January 24, 2020, some institutions had already launched a free crisis intervention hotline for the public (45). The government, academic societies, and hospitals quickly carried out cross-organizational cooperation, mobilized the resources they had, organized online training, organized academic conferences, and conducted telephone-based psychological interventions and internet-based psychological interventions for the public across China. Given that the fast transmission of the novel coronavirus between people may hinder traditional face-to-face psychological interventions and the widespread adoption of smartphones, online mental health services were heavily used in China. Massive use of online mental health services may maximize the beneficiaries from those programs, contributing to helping remove the barriers to accessing quality care for mental health.

Thirdly, the importance of implementation quality and outcome evaluation of those mental health interventions was emphasized. The Chinese government had emphasized in several guidelines that it was necessary to use qualified organizations and staff to provide psychological interventions, they also pointed out the importance of professional training (18, 24, 28, 31). Besides, the ethics of psychological intervention were highly valued in related guidelines (50). Also, the evaluation of the effectiveness of psychological interventions were emphasized (24), and the focus of psychological assistance was constantly being adjusted as the epidemic changed.

### Challenges of Mental Health Policies During the COVID-19 Outbreak

It is said that China has long been faced with an extremely low rate of mental health service utilization (58, 59). To date, most of the attention has been focused on the provision of mental health services, with the utilization of these services to a large extent neglected. He et al. (58, 60) reported that only 3.70–5.58% of the general population actively sought out mental health services during the COVID-19 outbreak. After a large increase in mental health services, how to improve the utilization of services is also an issue that needs to be urgently considered.

Due to the relatively short duration of the outbreak, many mental health policies released during the outbreak of COVID-19 had been implemented for only a few weeks, the implementation data found in the current review were limited. Based on the included data, it seems that most of mental health policies released during the COVID-19 outbreak were implemented, but the process and quality of implementation is still unclear. Although the importance of implementation quality and outcome evaluation of those mental health interventions was emphasized, relative data have not yet been published. Nationwide surveys of psychological well-being during the COVID-19 outbreak were conducted (61, 62), but those surveys did not describe whether they used mental health services. Another question is that most mental health services were provided via telephone and the internet. Both the implementation quality and program effectiveness are difficult to guarantee (9, 58). Thus, we think the integration of assessment in structure, process, and outcomes of those mental health programs is needed in the forthcoming months. These recommendations may inform how other countries can overcome the shortage of mental health resources when facing the COVID-19 outbreak. Moreover, people with low socioeconomic status (SES) may not be able to take full advantage of digital technologies that online mental health services rely on (46, 58). What should be considered is whether the uneven development of online mental health services for this epidemic will widen the mental health gap in China.

Furthermore, China currently lacks a well-established mental healthcare system, and has no existing national-level emergency response system and designated workforce to provide psychological crisis interventions during a national emergency or disaster (55). The shortage of professional human resources on mental health is also a problem. Over the past decade, the number of psychiatrists in China has increased (63). However, the amount of mental health professionals still cannot meet the needs of psychosocial intervention during this kind of large-scale epidemic (42). System defects and lack of personnel may have affected the implementation of mental health policies during the COVID-19 outbreak (64). In order to better respond to infectious disease outbreaks and ensure the effectiveness of mental health policies, it is important to address these issues in the future.

### Limitations

At first, the implementation data found in the current review were limited. It seems that most of the mental health policies released during the COVID-19 outbreak were implemented, but the process and quality of implementation is still unclear, which needs further exploration. Secondly, in order to presented as much data related to policy implementation as possible, we have included data from some comments and letters to the editor, which may not have been peer-reviewed. Another possible limitation is that this review is based on implemented mental health policies at the national level. Since all provinces in China are basically carrying out relevant work under the guidance of national-level mental health policies, we did not consider local-level mental health policies, there may be bias in the representativeness of the results.

### Implications for Future Research

Firstly, we think the long-term outcomes and implementation quality of these mental health interventions need further evaluation in future studies. Secondly, it is necessary to evaluate the impact of those known problems (such as low utilization of mental health services, with no existing national-level emergency response system and designated workforce) on the implementation of these mental health policies released during the outbreak of COVID-19. Besides, there was a lack of separate policies for specific mental health issues during the COVID-19 outbreak in China. Future research is needed to explore the necessity of making separate policies for some common mental health problems (such as PTSD) in a national emergency or disaster.

## Conclusions

This review suggests that China has responded quickly and comprehensively to the possible mental health crisis during the COVID-19 outbreak, appropriate mental health policies were released for different populations. As the epidemic situation continued to change, the focus of mental health policies was adjusted accordingly. However, we should note that there was a lack of separate policies for specific mental health issues during the COVID-19 outbreak. Such as PTSD, which is one of the most common mental health problems in a national emergency or disaster. In addition, the combination of telemedicine and face-to-face mental health services should be considered in middle-income and low-income countries, which may help remove barriers to accessing quality mental health care. Lastly, we believe this is a critical time to recognize these extraordinary advances in policy making, as it provides an unprecedented opportunity to evaluate the effects of mental health policies that China may adopt in the post-COVID-19 era.

## Data Availability Statement

The original contributions generated in the study are included in the article/Supplementary Materials, further inquiries can be directed to the corresponding author.

## Author Contributions

SX and DQ contributed to the design of the study. JH and FO searched through the databases. DQ and YL screened the text. DQ and LL extracted and analyzed the data. DQ wrote the first draft of the manuscript with input from SX. All the authors approved the final manuscript.

## Conflict of Interest

The authors declare that the research was conducted in the absence of any commercial or financial relationships that could be construed as a potential conflict of interest.

## Abbreviations

COVID-19: coronavirus disease 2019
SARS: severe acute respiratory syndrome
MERS-CoV: Middle East respiratory syndrome
Ebola: Ebola virus disease
WHO: World Health Organization
H1N1: 2009 influenza A(H1N1)
PTSD: post-traumatic stress disorder
SES: socioeconomic status.
