# Supplementary material for: Policies to Improve the Mental Health of People Influenced by COVID-19 in China: A Scoping Review
Source: Front Psychiatry. 2020 Dec 11;11:588137. doi: 10.3389/fpsyt.2020.588137 (PMC7759550; doi:10.3389/fpsyt.2020.588137)
Supplement: Supplementary file 1 [file Data_Sheet_1.PDF]

Search strategy
A: search strategy for published articles
Search strategy for PubMed:

**a) Mental health policy**	**Results**
1. national plan [Title/Abstract]	**759**
2. national program [Title/Abstract]	2150
3. national strategy [Title/Abstract]	**1133**
4. legislation [Title/Abstract]	**37302**
5. law [Title/Abstract]	**91720**
6. national reform [Title/Abstract]	**79**
7. health system [Title/Abstract]	**38871**
8. Health Policy [Title/Abstract]	**24650**
9. Mental health [Title/Abstract]	**155878**
10. Mental Health Policy [Title/Abstract]	**1009**
11. Mental Health Policies [Title/Abstract]	**300**
12. effect of Health Policy [Title/Abstract]	**413**
13. Policy Implementation [Title/Abstract]	**1733**
14. policy assessment [Title/Abstract]	**141**
15. Psychological assistant [Title/Abstract]	**477**
16. 1 OR 2 OR 3 OR 4 OR 5 OR 6 OR 7 OR 8 OR 9 OR 10 OR 11 OR 12 OR 13 OR 14 OR 15	**337008**
**b) COVID-19**	
17. COVID-19 [Title/Abstract]	**42920**
18. Coronavirus disease 2019 [Title/Abstract]	**88501**
19. Covid 19 [Title/Abstract]	**42920**

20. severe acute respiratory syndrome coronavirus 2 [Title/Abstract]	**4034**
21. SARS-CoV-2 [Title/Abstract]	**14157**
22. SARS-CoV [Title/Abstract]	**18803**
23. novel coronavirus [Title/Abstract]	**4792**
24. coronavirus [Title/Abstract]	**30535**
25. CoV-2 [Title/Abstract]	**16291**
26. 2019-nCoV [Title/Abstract]	**1123**
27. SARS COV2 [Title/Abstract]	**817**
28. 17 OR 18 OR 19 OR 20 OR 21 OR 22 OR 23 OR 24 OR 25 OR 26 OR 27	**59437**
**c) China**	
29. China [Title/Abstract]	**189450**
30. Chinese [Title/Abstract]	**213671**
31. 29 OR 30	**358042**
**d) a) AND b) AND c)**	
32. 16 AND 28 AND 31 AND ("2019/12/01"[Date - Publication] : "2020/05/20"[Date - Publication]))	**113**

Search strategy for Web of Science:

Search strategy	**Results**
**a) Mental health policy**	
TS=(“national plan” OR “national program” OR “national strategy” OR legislation OR law OR “national reform” OR “health system” OR “health policy” OR “mental health” OR “mental health policy” OR “mental health policies” OR “effect of health policy” OR “policy implementation” OR “policy assessment” OR “psychological assistant”)	**176571**
**b) COVID-19**	

TS=("COVID-19" OR "Coronavirus disease 2019" OR "severe acute respiratory syndrome coronavirus 2" OR "SARS-CoV-2" OR "SARS-CoV" OR "novel coronavirus" OR coronavirus OR "CoV-2" OR "2019-nCoV" OR "SARS COV2")	**54165**
**c) China**	
TS=(China OR Chinese)	**206432**
**d) a) AND b) AND c)**	**189**

Search strategy for CNKI:

Search strategy	**Results**
**b) Mental health policy**	
(AB='国家计划'+'国家项目'+'国家策略'+'法律'+'卫生政策'+'精神卫生政策'+'心理援助')	**54810**
**b) COVID-19**	
(AB='COVID-19'+'新冠肺炎'+'冠状病毒'+' SARS-CoV-2'+'Covid-19')	**67391**
**c) China**	
(AB='中国'+'中国人')	**384518**
**d) a) AND b) AND c)**	**53**

B Search strategy for grey literature

Keywords such as"policy", "national plan", "national program", "COVID-19", "SARS-CoV-2" were combined with "mental health" in our search.

Database	Kedwords	Results
State Council Policy Document Database of China (http://www.gov.cn/index.htm) ("2019/12/01"[Date - Publication] : "2020/05/20"[Date - Publication]))	新冠肺炎	171

	新型冠状病毒性肺炎	171
	心理健康	27
	心理危机干预	9
	心理援助	13
	Finally, a total of 171 different references were screend	171
National science and technology library of China ("2019/12/01"[Date - Publication] : "2020/05/20"[Date - Publication]))	‘新冠肺炎’ and ‘心理健康’	19
	‘新冠肺炎’ and ‘心理援助’	8
	‘新冠肺炎’ and ‘精神卫生政策’	0
	‘新冠肺炎’ and ‘卫生政策’	2
	Finally, a total of 20 different references were screend	20
National Library of China ("2019/12/01"[Date - Publication] : "2020/05/20"[Date - Publication]))	新冠肺炎	11
	新型冠状病毒性肺炎	0
	‘新冠肺炎’ and ‘心理健康’	1
	‘新冠肺炎’ and ‘心理援助’	1
	‘新冠肺炎’ and ‘精神卫生政策’	0
	‘新冠肺炎’ and ‘卫生政策’	0
	Finally, a total of 10 different references were screend	10
Chinese Association for Mental Health (http://www.camh.org.cn/) ("2019/12/01"[Date - Publication] : "2020/05/20"[Date - Publication]))	新冠肺炎	21
	新型冠状病毒性肺炎	3

	心理健康	18
	心理危机干预	2
	心理援助	5
	Finally, a total of 23 different references were screend	23
Chinese Psychological society (https://www.cpsbeijing.org/) ("2019/12/01"[Date - Publication] : "2020/05/20"[Date - Publication]))	新冠肺炎	78
	新型冠状病毒性肺炎	35
	心理健康	47
	心理危机干预	33
	心理援助	29
	Finally, a total of 92 different references were screend	92

Table S1

Preferred Reporting Items for Systematic reviews and Meta-Analyses extension for Scoping Reviews (PRISMA-ScR) Checklist

SECTION	ITEM	PRISMA-ScR CHECKLIST ITEM	REPORTED ON PAGE AND LINE #
**TITLE**			
Title	1	Identify the report as a scoping review.	Page 1<br>Line 1-2
**ABSTRACT**			
Structured summary	2	Provide a structured summary that includes (as applicable): background, objectives, eligibility criteria, sources of evidence, charting methods, results, and conclusions that relate to the review questions and objectives.	Page 1<br>Line 31-66
**INTRODUCTION**			
Rationale	3	Describe the rationale for the review in the context of what is already known. Explain why the review questions/objectives lend themselves to a scoping review approach.	Page 2<br>Line 92-126
Objectives	4	Provide an explicit statement of the questions and objectives being addressed with reference to their key elements (e.g., population or participants, concepts, and context) or other relevant key elements used to conceptualize the review questions and/or objectives.	Page 4-5<br>Line 108-126; 138-145
**METHODS**			

SECTION	ITEM	PRISMA-ScR CHECKLIST ITEM	REPORTED ON PAGE AND LINE #
Protocol and registration	5	Indicate whether a review protocol exists; state if and where it can be accessed (e.g., a Web address); and if available, provide registration information, including the registration number.	N/A
Eligibility criteria	6	Specify characteristics of the sources of evidence used as eligibility criteria (e.g., years considered, language, and publication status), and provide a rationale.	Page 5<br>Line 129-163
Information sources*	7	Describe all information sources in the search (e.g., databases with dates of coverage and contact with authors to identify additional sources), as well as the date the most recent search was executed.	Page 5<br>Line 147-163
Search	8	Present the full electronic search strategy for at least 1 database, including any limits used, such that it could be repeated.	Supplementary materials
Selection of sources of evidence†	9	State the process for selecting sources of evidence (i.e., screening and eligibility) included in the scoping review.	Page 8<br>Line 215-221; Fig. 1
Data charting process‡	10	Describe the methods of charting data from the included sources of evidence (e.g., calibrated forms or forms that have been tested by the team before their use, and whether data charting was done independently or in duplicate) and any processes for obtaining and confirming data from investigators.	Page 7<br>Line 194-212
Data items	11	List and define all variables for which data were sought and any assumptions and simplifications made.	Page 7<br>Line 194-206
Critical appraisal of	12	If done, provide a rationale for conducting a critical appraisal of included sources of evidence; describe	Page 7

SECTION	ITEM	PRISMA-ScR CHECKLIST ITEM	REPORTED ON PAGE AND LINE #
individual sources of evidence§		the methods used and how this information was used in any data synthesis (if appropriate).	Line 209-212<br>Supplementary materials
Synthesis of results	13	Describe the methods of handling and summarizing the data that were charted.	Page 7<br>Line 201-206
**RESULTS**			
Selection of sources of evidence	14	Give numbers of sources of evidence screened, assessed for eligibility, and included in the review, with reasons for exclusions at each stage, ideally using a flow diagram.	Page 8<br>Line 215-221; Fig. 1
Characteristics of sources of evidence	15	For each source of evidence, present characteristics for which data were charted and provide the citations.	Page 8<br>Line 222-233
Critical appraisal within sources of evidence	16	If done, present data on critical appraisal of included sources of evidence (see item 12).	Page 8<br>Line 231-233; Table S3
Results of individual sources of evidence	17	For each included source of evidence, present the relevant data that were charted that relate to the review questions and objectives.	15, 16
Synthesis of results	18	Summarize and/or present the charting results as they relate to the review questions and objectives.	6-8, 18-21

SECTION	ITEM	PRISMA-ScR CHECKLIST ITEM	REPORTED ON PAGE AND LINE #
**DISCUSSION**			
Summary of evidence	19	Summarize the main results (including an overview of concepts, themes, and types of evidence available), link to the review questions and objectives, and consider the relevance to key groups.	Page 8-11<br>Line 234-321;<br>table 2, table 3
Limitations	20	Discuss the limitations of the scoping review process.	Page 15<br>Line 421-431
Conclusions	21	Provide a general interpretation of the results with respect to the review questions and objectives, as well as potential implications and/or next steps.	Page 16-17<br>Line 446-458
**FUNDING**			
Funding	22	Describe sources of funding for the included sources of evidence, as well as sources of funding for the scoping review. Describe the role of the funders of the scoping review.	Page 16<br>Line 472-475

Table S2 JBI Critical appraisal checklist for narrative, expert opinion & text.

1.	Is the source of the opinion cleanly identified?
2.	Does the source of the opinion have standing in the field of expertise?
3.	Are the interest of patients/clients the central focus of the opinion?
4.	Is the opinion's basis in logic/experience clearly argued?
5.	Is the argument developed analytical?
6.	Is there reference to the extant literature/ evidence and any incongruency with it logically defended?
7.	Is the opinion supported by peers?

Table S3 Critical appraisal checklist for the included studies.

Study	1	2	3	4	5	6	7	Assessment
Miu et al. (9)	Y	Y	Y	Y	Y	Y	U	Adequate
Li et al. (10)	Y	Y	Y	Y	Y	Y	Y	Strong
Liu et al. (11)	Y	Y	Y	Y	Y	Y	U	Adequate
Chinese Psychological Society (37)	Y	Y	Y	NA	NA	N	U	Weak
Chinese Psychological Society (38)	Y	Y	Y	Y	Y	U	U	Adequate
Wang et al. a (39)	Y	Y	Y	Y	Y	Y	U	Adequate
Zhou et al. (40)	Y	Y	Y	Y	Y	Y	U	Adequate
Zhang et al. (41)	Y	Y	Y	Y	Y	Y	U	Adequate
Yang et al. (42)	Y	Y	Y	Y	Y	Y	U	Adequate
Wang et al. b (43)	Y	Y	Y	Y	Y	Y	U	Adequate
Kang et al. a (44)	Y	Y	Y	Y	Y	Y	U	Adequate
Kang et al. b (45)	Y	Y	Y	Y	Y	Y	U	Adequate

Hu et al. (46)	Y	Y	Y	Y	Y	Y	U	Adequate
Cui et al. (47)	Y	Y	Y	Y	Y	Y	U	Adequate
Chen et al. (48)	Y	Y	Y	N	Y	N	U	Adequate
National Health Commission of China (49)	Y	Y	Y	NA	NA	NA	Y	Weak
Chinese Psychological Society (50)	Y	Y	Y	NA	NA	NA	NA	Weak
Chinese Psychological Society (51)	Y	Y	Y	NA	NA	NA	NA	Weak
Song et al (52)	Y	Y	Y	Y	Y	Y	U	Adequate
Zang et al (53)	Y	Y	Y	Y	Y	Y	Y	Strong
Ju et al (54)	Y	Y	Y	Y	Y	Y	Y	Strong

Note: (Y=Yes, N=No, NA=not applicable, U=unclear) (Strong, adequate, weak, unknown)
